# Detection of circulating tumour DNA is associated with inferior outcomes in Ewing sarcoma and osteosarcoma: a report from the Children’s Oncology Group

**DOI:** 10.1038/s41416-018-0212-9

**Published:** 2018-08-21

**Authors:** David S. Shulman, Kelly Klega, Alma Imamovic-Tuco, Andrea Clapp, Anwesha Nag, Aaron R. Thorner, Eliezer Van Allen, Gavin Ha, Stephen L. Lessnick, Richard Gorlick, Katherine A. Janeway, Patrick J. Leavey, Leo Mascarenhas, Wendy B. London, Kieuhoa T. Vo, Kimberly Stegmaier, David Hall, Mark D. Krailo, Donald A. Barkauskas, Steven G. DuBois, Brian D. Crompton

**Affiliations:** 1 Dana-Farber/Boston Children’s Cancer and Blood Disorders Center, Harvard Medical School, Boston, MA USA; 2 Center for Cancer Genome Discovery, Dana-Farber Cancer Institute, Boston, MA USA; 3 Department of Medical Oncology, Dana-Farber Cancer Institute, Boston, MA USA; 4 Broad Institute, Cambridge, MA USA; 5 Center for Childhood Cancer and Blood Diseases at Nationwide Children’s Hospital Research Institute and the Division of Pediatric Heme/Onc/BMT at The Ohio State University, Columbus, OH USA; 6 Department of Pediatrics, MD Anderson Cancer Center, Houston, TX USA; 7 Department of Pediatrics, University of Texas Southwestern Medical Center at Dallas, Dallas, TX USA; 8 Division of Hematology, Oncology, and Blood and Marrow Transplantation, Children’s Hospital Los Angeles, Keck School of Medicine, University of Southern California, Los Angeles, CA USA; 9 Department of Pediatrics, UCSF Benioff Children’s Hospital, University of California, San Francisco School of Medicine, San Francisco, CA USA; 10 Children’s Oncology Group, Monrovia, CA USA; 11 Department of Preventative Medicine, Keck School of Medicine, University of Southern California, Los Angeles, CA USA; 12 Present Address: Department of Pediatric Oncology, 450 Brookline Avenue, Boston, MA USA

**Keywords:** Tumour biomarkers, Sarcoma, Bone cancer

## Abstract

**Background:**

New prognostic markers are needed to identify patients with Ewing sarcoma (EWS) and osteosarcoma unlikely to benefit from standard therapy. We describe the incidence and association with outcome of circulating tumour DNA (ctDNA) using next-generation sequencing (NGS) assays.

**Methods:**

A NGS hybrid capture assay and an ultra-low-pass whole-genome sequencing assay were used to detect ctDNA in banked plasma from patients with EWS and osteosarcoma, respectively. Patients were coded as positive or negative for ctDNA and tested for association with clinical features and outcome.

**Results:**

The analytic cohort included 94 patients with EWS (82% from initial diagnosis) and 72 patients with primary localised osteosarcoma (100% from initial diagnosis). ctDNA was detectable in 53% and 57% of newly diagnosed patients with EWS and osteosarcoma, respectively. Among patients with newly diagnosed localised EWS, detectable ctDNA was associated with inferior 3-year event-free survival (48.6% vs. 82.1%; *p* = 0.006) and overall survival (79.8% vs. 92.6%; *p* = 0.01). In both EWS and osteosarcoma, risk of event and death increased with ctDNA levels.

**Conclusions:**

NGS assays agnostic of primary tumour sequencing results detect ctDNA in half of the plasma samples from patients with newly diagnosed EWS and osteosarcoma. Detectable ctDNA is associated with inferior outcomes.

## Introduction

Ewing sarcoma and osteosarcoma are the most common bone malignancies of childhood and adolescence. Approximately 70–75% of patients with either localised Ewing sarcoma or osteosarcoma are expected to survive their disease with multiagent chemotherapy regimens and local control of the primary tumour.^1–4^ While a range of clinical prognostic factors (e.g., tumour site and response to therapy) have been evaluated in these diseases,^5–7^ identification of the 25–30% of patients with localised disease with inadequate outcomes remains challenging.

Development of circulating prognostic biomarkers in patients with localised disease is a high priority. Ewing sarcoma is characterised by hallmark translocation events (most commonly *EWSR1/FLI1*). Prior studies have evaluated the prognostic impact of fusion transcript detection in the peripheral blood or bone marrow using reverse transcription-polymerase chain reaction, yet have not shown consistent prognostic value.^8,9^ Likewise, measures of Ewing sarcoma tumour cells in the peripheral blood and bone marrow by flow cytometry were not prognostic.^9–11^ A range of circulating biomarkers have been evaluated in osteosarcoma, though none yet validated.^12–15^

Circulating tumour DNA (ctDNA)-based assays hold promise as potentially important peripheral biomarkers. Successful utilisation of ctDNA for disease prognostication and association with response to therapy in patients with carcinomas has relied on the identification of highly recurrent single-nucleotide variants (SNVs).^16^ Pediatric solid tumours are less amenable to such approaches, because these malignancies lack recurrent SNVs. Ewing sarcoma and osteosarcoma are at opposite ends of the spectrum of cancer genomic complexity. Ewing sarcoma is characterised by a simple translocation-driven genome, with *STAG2* and *TP53* loss-of-function mutations found in a minority of tumours.^17–19^ The majority of Ewing sarcoma tumours express an *EWSR1/ETS* translocation with a patient-specific intronic breakpoint, precluding the use of an assay that detects a single breakpoint across patients. Prior groups have detected ctDNA in patients with Ewing sarcoma using patient-specific digital droplet PCR (ddPCR) or hybrid capture next-generation sequencing (NGS).^20–22^ In this study, we utilised a custom hybrid capture NGS assay, termed TranSS-Seq, which we previously validated to detect ctDNA from patients with Ewing sarcoma.^23^

The osteosarcoma genome is characterised by complex translocations and copy number changes. 8q gains are relatively common, may reflect *MYC* copy number gain/amplification, and may confer an inferior prognosis.^24^ Prior attempts to identify ctDNA in the peripheral blood of patients with osteosarcoma utilised tumour biopsy sequencing to create probes for ctDNA detection and targeted NGS of commonly mutated genes.^25,26^ Ultra-low-pass whole-genome sequencing (ULP-WGS) is a NGS method capable of identifying the complex structural variants seen in osteosarcoma.^27^ These divergent patterns of genomic aberration (translocation associated vs. complex structural changes) are common in pediatric malignancies and provide potential avenues for detection of ctDNA.

In this context, we conducted a retrospective cohort study to evaluate two NGS ctDNA methods capable of ctDNA detection without available tumour sequencing in patients with these diseases. We hypothesised that ctDNA would be detectable in blood samples and that the presence and level of ctDNA would be associated with clinical outcomes in patients with newly diagnosed localised disease. When possible, we controlled for previously described clinical features associated with outcomes in these diseases. Finally, we leveraged these techniques to study additional tumour characteristics (*STAG2* and *TP53* mutations in Ewing sarcoma; 8q gain in osteosarcoma) in ctDNA.

## Methods

### Patient eligibility and sample collection

#### Ewing sarcoma cohort

Patients in the Ewing sarcoma cohort were required to have a pathologic diagnosis of Ewing sarcoma and be enrolled on the COG Ewing sarcoma biology study AEWS07B1. Patients included in the primary analysis were required to have newly diagnosed localised disease. Samples from patients who presented with newly diagnosed metastatic disease or recurrent disease were analysed as separate cohorts. For each patient, ctDNA was analysed from a single blood sample drawn within 28 days of diagnosis or relapse and prior to the start of therapy. Each participating centre obtained a blood sample in an EDTA tube that was shipped overnight at room temperature to the University of California, San Francisco. On arrival, all samples were centrifuged, plasma was isolated, and then frozen at −70 °C until ctDNA analysis. The median plasma volume for this cohort was 2 mL (range, 0.75–2.0).

#### Osteosarcoma cohort

Patients in the osteosarcoma cohort were required to have a new pathologic diagnosis of localised osteosarcoma and were enrolled on the COG osteosarcoma biology study AOST06B1. Each patient had a blood sample collected in an EDTA tube prior to the start of therapy. Plasma was isolated on-site and frozen at −70 °C before being shipped to Nationwide Children’s for storage prior to ctDNA analysis. The median plasma volume for this cohort was 2 mL (range, 0.75–6.75).

#### Both cohorts

All patients signed written informed consent at the time of enrollment to AEWS07B1 or AOST06B1. Separate approval for this retrospective use of patient samples and clinical data was obtained from the Dana-Farber/Harvard Cancer Center institutional review board.

### ctDNA analysis

Cell-free DNA was extracted from plasma samples using the QIAamp Circulating Nucleic Acid Kit (Qiagen). Quantification of total cell-free DNA in ng/mL was performed using Quant-iT PicoGreen dsDNA Assay Kit (Thermo Fisher Scientific). Contamination of the sample with high-molecular weight DNA was determined by Bioanalyzer (Agilent) and an SPRI clean-up was performed to select for ctDNA if necessary. In total, 37 (39%) samples from patients with Ewing sarcoma and 2 (3%) patients with osteosarcoma underwent SPRI clean-up. Up to 40 ng of DNA were used for library preparation using the KAPA Hyper Prep Kit (Kapa Biosystems). Barcoded adapters were ligated during manual library preparation. Libraries were assessed by Bioanalyzer and quantified for pooling using the MiSeq Nano flow cell.

For detection of Ewing translocations, sequencing libraries were enriched using the Agilent SureSelectXT Hybrid Capture Kit and a validated custom bait set targeting intronic regions of genes commonly involved in sarcoma translocations, including *EWSR1*, *FUS*, *CIC*, and CCNB3 and the coding regions of *TP53* and *STAG2*. This approach, termed TranSS-Seq, allows for the detection of translocations involving these genes and any translocation partner as well as coding mutations in *TP53* and *STAG2*. Post-enrichment libraries were quantified and sequenced with intended unique coverage at target regions of >500×. The average measured coverage at enrichment sites for all samples tested by this approach was 579× (range 151.8–1311.2×).

For ULP-WGS for osteosarcoma samples, barcoded sequencing libraries were pooled and sequenced on an Illumina HiSeq 2500 to achieve an anticipated average coverage between 0.2× and 1× for the whole human genome.

Samples for both TranSS-Seq and ULP-WGS were de-multiplexed, aligned, and processed using Picard tools, BWA alignment tool, and GATK tool.^28–30^ Identification of targeted translocations by TranSS-Seq was performed using BreaKmer.^31^ To quantify the number of translocation reads and wild-type reads for every sample, each sequencing read was realigned to either the human reference genome or a custom sequence containing the patient-specific *EWSR1/ETS* translocation based on sequence homology. Percent ctDNA was calculated based on the expectation that each cancer genome contains one translocated and one wild-type *EWSR1* allele, while normal genomes contain two wild-type *EWSR1* alleles: % ctDNA = *T*/(((*W* − *T*)/2) + *T*), where *T* is the number of translocation reads and *W* is the number of wild-type reads. In a previous study, we demonstrated that ctDNA levels determined with TranSS-Seq were highly correlated with experimental serial dilution experiments and levels measured by patient-specific ddPCR. We also demonstrated that TranSS-Seq has an estimated sensitivity for detection of Ewing sarcoma ctDNA levels at or below 1.5% of total cell-free DNA.^23^

ULP-WGS analysis was performed using the Broad Institute’s ichorCNA algorithm, with manual curation of results to confirm tumour percentages.^27^ Previous studies demonstrate that ULP-WGS can be used to identify ctDNA in patients with copy number-altered tumours. Serial dilution experiments validated that this approach can detect and accurately quantify ctDNA when constituting as little as 3% of a cell-free DNA sample.^23^

### Independent variables

The primary independent variable was ctDNA coded as positive or negative ('ctDNA positivity') for detectable fusion ctDNA in the Ewing sarcoma cohort or detectable copy number alterations in the osteosarcoma cohort. Percent ctDNA and total cell-free DNA (ng/mL) were analysed as separate continuous variables and provided a secondary independent variables for analysis.

### Dependent variables

The following variables obtained from AEWS07B1 and AOST06B1 were used to characterise patients: age at study enrollment; sex; whether the sample was drawn at the time of initial diagnosis or at relapse (relevant for Ewing sarcoma cohort only); stage; primary site; and vital status. Age was dichotomised for multivariate analysis to <18 or ≥18 for patients with Ewing sarcoma and <14 or ≥14 for patients with osteosarcoma.^6,32^ Tumour size was measured as largest diameter in a subset of patients with Ewing sarcoma treated on a COG clinical trial (NCT01231906) and dichotomised according to established prognostic size criteria of <8 cm or ≥8 cm.^1^ The primary endpoint was event-free survival (EFS) and was defined as time from enrollment to first episode of disease progression, second malignancy, or death, with patients without event censored at last follow-up. Overall survival (OS) was defined as time from enrollment to death, with alive patients censored at last follow-up

### Statistical analysis

This binary predictor variable 'ctDNA positivity' was tested for association with clinical and demographic features using Fisher's exact tests and *t* tests as appropriate. Cell-free DNA quantities were tested for association with clinical features using Wilcoxon's rank-sum tests. Cell-free DNA was analysed as a continuous variable and tested for correlation with percent ctDNA using the Spearman's correlation coefficient. EFS and OS were estimated by Kaplan–Meier methods with 95% confidence intervals (CIs). Potential associations between EFS or OS with ctDNA positivity were tested with log-rank tests. We used Cox proportional hazards models of EFS and OS to assess the prognostic impact of the continuous ctDNA and cell-free DNA secondary predictors and to assess prognostic impact of ctDNA positivity independent of other prognostic factors in these diseases. A global test of proportional hazards was used to confirm the proportional hazards assumption. All *p* values are two-sided and a *p* value < 0.05 was considered statistically significant. All statistical analyses were performed with Stata^®^.

## Results

### Patient characteristics

We analysed ctDNA in 100 blood samples from 98 unique patients with Ewing sarcoma. Samples not drawn at the time of diagnosis or relapse (*n* = 4) and subsequent samples drawn from the same patient with a sample from an earlier timepoint (*n* = 2) were excluded. The Ewing sarcoma analytical cohort therefore included 94 patients (Table 1).

**Table 1:** Characteristics of 94 patients with Ewing sarcoma and 72 patients with osteosarcoma with available ctDNA results

	Ewing sarcoma cohort (*n* = 94)	Osteosarcoma cohort (*n* = 72)
*Sex*		
Male	53 (56.4)	46 (63.9)
Female	41 (43.6)	26 (36.1)
Age at diagnosis, years (median, range)	14.1 (1.9–20.6)^a^	13.6 (5.6–22.4)
*Primary disease site*		
Pelvic	20 (26.3)^b^	
Non-pelvic	56 (73.7)	
Femur		31 (43.1)
Tibia		24 (33.3)
Humerus		8 (11.1)
Fibula		5 (6.9)
Other		4 (5.6)
*Stage at diagnosis*		
Localised	52 (63.4)^c^	72 (100)
Metastatic	30 (36.6)	0 (0)
*Time of sample acquisition*		
Initial diagnosis	77 (81.9)	72 (100)
Relapse	17 (18.1)	0 (0)

^a^Age at diagnosis available for 77 patients with newly diagnosed disease.

^b^Primary site data available for 76 patients with Ewing sarcoma.

^c^Stage at diagnosis available for 82 patients with Ewing sarcoma

We analysed ctDNA in 75 blood samples from 75 unique patients with newly diagnosed, localised osteosarcoma, each with a single blood sample taken at the time of diagnosis. Three patients presented with osteosarcoma as a secondary malignancy and were excluded a priori. The osteosarcoma analytical cohort therefore included 72 patients with primary osteosarcoma (Table 1).

### ctDNA is detectable at the time of diagnosis and relapse in patients with bone malignancies

Within the Ewing sarcoma cohort, we detected ctDNA in 53.3% (41/77) of newly diagnosed patients and 47.1% (8/17) of patients with relapsed disease (*p* = 0.79; Table 2). Among patients with newly diagnosed Ewing sarcoma with detectable ctDNA, the median percent of total cell-free DNA that was ctDNA containing an *EWSR1* translocation was 13.8% (range 1.4–43.2%). The median quantity of total cell-free DNA was 14.2 ng/mL (range 2.4–255.3). There was a weak correlation between total cell-free DNA and percent ctDNA (Supplemental Fig. 1).

**Table 2:** Association between ctDNA detection and clinical features in patients with Ewing sarcoma and osteosarcoma

	ctDNA positive	ctDNA negative	*p* value
*Ewing sarcoma (n = 94)*			
Full cohort	49 (52.1)	45 (47.9)	
Initial diagnosis (*n* = 77)	41 (53.3)^a^	36 (46.8)	0.79
Relapse (*N* = 17)	8 (47.1)	9 (52.9)	
Initial diagnosis (*n* = 77)			
Age (mean, 95% CI)	13.4 (12.1–14.8)	13.3 (11.8–14.8)	0.85
Male	23 (54.8)	19 (45.2)	0.82
Female	18 (51.4)	17 (48.6)	
Metastatic	18 (69.2)	8 (30.8)	0.053
Non-metastatic	22 (44.0)	28 (56.0)	
Pelvic primary	12 (66.7)	6 (33.3)	0.18
Non-pelvic primary	25 (46.3)	29 (53.7)	
Tumour size <8 cm	5 (33.3)	10 (66.7)	0.063
Tumour size ≥8 cm	5 (83.3)	1 (16.7)	
*Osteosarcoma (n* *=* *72)*			
Full cohort	41 (56.9)	31 (43.1)	
Age (mean, 95% CI)	14.0 (12.9–15.2)	12.9 (11.4–14.3)	0.2
Male	25 (54.4)^a^	21 (45.7)	0.63
Female	16 (61.5)	10 (38.5)	
Femur primary	22 (71.0)	9 (29.0)	0.054
Other primary	19 (46.3)	22 (53.7)	

^a^Percentages do not equal 100% due to rounding

The 49 positive samples included *EWSR1/FLI1* (*n* = 43), *EWSR1/ERG* (*n* = 5), and one novel *EWSR1/CSMD2* fusion. This novel fusion has not previously been described and we therefore obtained tumour material and confirmed the presence of this fusion using PCR (Supplemental Methods and Supplemental Fig. 2).

Within the osteosarcoma cohort, 56.9% (41/72) had detectable ctDNA. Among patients with osteosarcoma with detectable ctDNA, the median percent of total cell-free DNA that was ctDNA was 11% (range 4.6–58%). The median quantity of total cell-free DNA was 4.5 ng/mL (range 1.7–318.2). There was a weak correlation between total cell-free DNA and percent ctDNA (Supplemental Fig. 3).

### Detection of ctDNA is associated with clinical features in Ewing sarcoma and osteosarcoma

We compared binary detection of ctDNA with presenting patient and tumour characteristics (Table 2). Among patients with newly diagnosed Ewing sarcoma, ctDNA was detected in 69.2% of patients with metastatic disease compared to 44.0% of patients with localised disease (*p* = 0.053). ctDNA was detected in 66.7% of patients with newly diagnosed pelvic Ewing sarcoma compared to 46.3% of patients with non-pelvic Ewing sarcoma (*p* = 0.18). Among patients with tumour size collected (*n* = 21), 83.3% of patients with tumour size ≥8 cm maximum diameter had detectable ctDNA compared to 33.3% of patients with tumour size <8 cm maximum diameter (*p* = 0.063, Table 2). In the osteosarcoma cohort, 71.0% of patients with femoral primary tumours had detectable ctDNA compared to 46.3% of patients with tumours originating from other sites (*p* = 0.054). Total cell-free DNA was higher among patients with newly diagnosed pelvic Ewing sarcoma compared to non-pelvic sites, but no other significant associations were seen between total cell-free DNA and clinical features in either Ewing sarcoma or osteosarcoma (Supplemental Tables 1 and 2).

### Presence of detectable ctDNA is associated with inferior outcomes in Ewing sarcoma

**Fig. 1:**
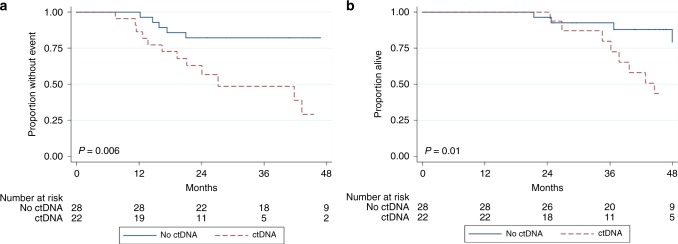
**a** EFS by ctDNA detection in patients with newly diagnosed localised Ewing sarcoma. **b** Overall survival by ctDNA detection in patients with newly diagnosed localised Ewing sarcoma

Clinical outcome data and ctDNA results were available for 50 patients with newly diagnosed localised Ewing sarcoma (median follow-up 41 months). Patients with detectable ctDNA had inferior EFS and OS (Fig. 1). The 3-year EFS and OS estimates in patients with detectable ctDNA were 48.6% (95% CI, 24.2–69.4) and 79.8% (95% CI, 49.4–93.0) compared to 82.1% (95% CI, 62.3–92.2) and 92.6% (95% CI, 73.4–98.1) in those without detectable ctDNA (*p* = 0.006 and 0.0125), respectively. In multivariate analyses, ctDNA positivity remained prognostic of EFS and OS after controlling for age and pelvic site (Table 3).

**Table 3:** Association between ctDNA detection and outcomes in patients with newly diagnosed localised Ewing sarcoma and osteosarcoma

	HR if ctDNA detected	95% CI	*p* value
*Ewing sarcoma (n* *=* *50)*			
Unadjusted EFS	3.74	1.4–10.1	0.009
Adjusted EFS^a^	3.85	1.4–10.9	0.011
Unadjusted OS	4.02	1.2–13.1	0.021
Adjusted OS^a^	3.76	1.06–13.3	0.04
*Osteosarcoma (n* *=* *72)*			
Unadjusted EFS	1.95	0.7–5.08	0.17
Adjusted EFS^b^	2.26	0.9–5.9	0.098
Unadjusted OS	3.96	0.9–18.1	0.076
Adjusted OS^b^	4.15	0.9–19.0	0.066

^a^Adjusted for age <18 vs. age ≥18 years and pelvic primary site. Disease site available for 49 patients.

^b^Adjusted for age <14 vs. age ≥14 years and sex

We analysed percent ctDNA as a continuous variable among patients with newly diagnosed localised Ewing sarcoma. The EFS and OS hazard ratios (HRs) for each unit increase in percent ctDNA were 1.06 (95% CI, 1.02–1.09; *p* = 0.002) and 1.06 (95% CI, 1.02–1.11; *p* = 0.003), respectively. When limiting the analysis only to those patients with detectable ctDNA (*n* = 22), the analogous HRs were 1.04 (95% CI, 0.99–1.09; *p* = 0.085) and 1.05 (95% CI, 0.99–1.11; *p* = 0.12). Among patients with localised Ewing sarcoma, higher cell-free DNA levels were only associated with an inferior OS (HR 1.04, 95% CI, 1.01–1.08; *p* = 0.01; Supplemental Table 1).

Clinical outcome data and ctDNA results were available for 23 patients with newly diagnosed metastatic Ewing sarcoma. Patients in this group with detectable ctDNA had inferior EFS compared to patients with no detectable ctDNA (3-year EFS: 34.1% (95% CI, 12.6–57.2; *n* = 8) vs. 85.7% (95% CI, 33.4–97.9; *n* = 18); *p* = 0.05; Supplemental Figure 4A). The observed difference in OS according to ctDNA positivity in this cohort was not statistically significant (*p* = 0.24, Supplemental Figure 4B).

### ctDNA level is associated with inferior outcomes in osteosarcoma

**Fig. 2:**
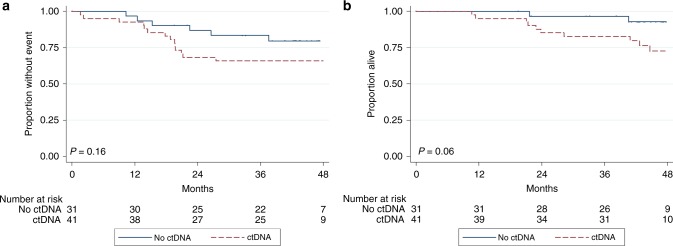
**a** EFS by ctDNA detection in patients with newly diagnosed localised osteosarcoma. **b** Overall survival by ctDNA detection in patients with newly diagnosed localised osteosarcoma

Clinical outcome data and ctDNA results were available for 72 patients with newly diagnosed localised osteosarcoma (median follow-up 44.3 months). EFS and OS estimates were numerically lower for patients with detectable ctDNA, but these differences were not statistically significant (Fig. 2). After controlling for age (≥14 or <14 years) and sex, two variably reported prognostic factors in osteosarcoma,^6,7^ ctDNA detection remained positively associated with inferior EFS and OS, but the results were also not statistically significant (Table 3).

Evaluating percent ctDNA as a continuous variable, the HRs for each unit increase in percent ctDNA among patients with localised osteosarcoma (*n* = 72) were 1.06 (95% CI, 1.03–1.09; *p* < 0.001) and 1.09 (95% CI, 1.06–1.14; *p* < 0.001) for EFS and OS, respectively. When limiting the analysis to the 41 patients with detectable ctDNA, the analogous HRs were 1.07 (95% CI, 1.036–1.11; *p* < 0.001) and 1.10 (95% CI, 1.05–1.16; *p* < 0.001). Among all patients with newly diagnosed osteosarcoma, cell-free DNA levels were not associated with clinical outcomes (Supplemental Table 2).

### Identification of genetic features of Ewing sarcoma and osteosarcoma via ctDNA

We attempted to determine whether potentially prognostic genetic features could be detected in ctDNA in patients with Ewing sarcoma and osteosarcoma. We were able to detect loss-of-function *STAG2* mutations in three patients and *TP53* mutations in four patients. The allelic fraction of these mutations correlated with the % ctDNA levels observed in the patient sample suggesting these are likely somatic events. Furthermore, as germline *STAG2* loss-of-function mutations have not been described, these mutations are expected to be somatic. Although germline *TP53* mutations in Ewing sarcoma are rare,^33^ we cannot definitively confirm that these events were somatic in the absence of germline DNA.

**Fig. 3:**
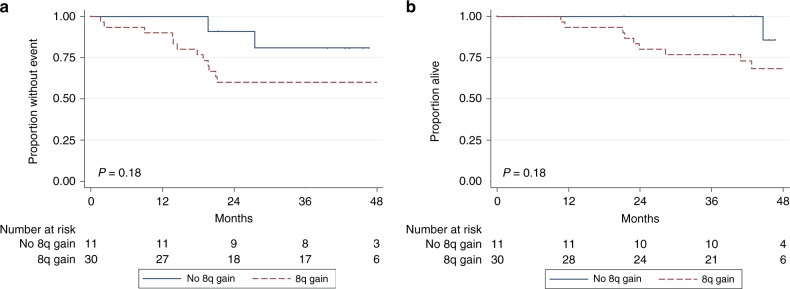
**a** EFS by 8q gain among patients with newly diagnosed localised osteosarcoma and detectable ctDNA. **b** Overall survival by 8q gain among patients with newly diagnosed localised osteosarcoma and detectable ctDNA

In osteosarcoma, we focused on 8q gain as a specific biological feature of interest. Among the 41 patients with detectable ctDNA in the osteosarcoma cohort, 8q gain was detected in 74.4% (32/43). The 3-year EFS for patients with 8q gain (*n* = 32) in ctDNA was 60.0% (95% CI, 40.5–75.0) compared to 80.9 (95% CI, 42.4–94.9) in patients without 8q gain (*n* = 11) in ctDNA (*p* = 0.18; Fig. 3).

## Discussion

Using two NGS ctDNA assays, we detected ctDNA in banked peripheral blood samples from 52.1% of patients with Ewing sarcoma and 56.9% of patients with osteosarcoma, all without the knowledge of tumour tissue sequencing results. Detectable ctDNA showed trends toward significant associations with metastatic disease and tumour size in Ewing sarcoma and with femoral primary site in osteosarcoma. Among patients with newly diagnosed localised Ewing sarcoma, binary detection of ctDNA was associated with an inferior outcome. In both diseases, an increased risk of event and death was significantly associated with an increase in ctDNA burden when evaluated as a continuous variable, a finding that was not seen when using total cell-free DNA as the marker of interest. Our study is therefore the first large study to demonstrate that qualitative and quantitative ctDNA detection provides prognostic information for patients with localised bone tumours. Finally, we identified additional genomic features from ctDNA including identification of a previously undescribed *EWSR1* fusion, *STAG2* loss in Ewing sarcoma, and 8q gain in osteosarcoma. Our results demonstrate that these two ctDNA assays may yield additional information about tumour biology.

While ctDNA analysis has proven useful for patients with carcinomas, there have been relatively few studies evaluating the utility of ctDNA as a biomarker in patients with sarcomas. Four studies have demonstrated that detection of ctDNA using ddPCR or hybrid capture-based NGS is feasible in Ewing sarcoma but each effort had too few patients to demonstrate a prognostic value of these assays.^20–23^ A similar approach has been utilised in a small cohort of patients with chondrosarcoma,^34^ and two studies including patients with osteosarcoma.^25,26^ Our study demonstrates the feasibility of detecting ctDNA naïve of the tumour genome. Leveraging thematic genome alterations in these two genomically diverse tumours provided an avenue to efficiently detect ctDNA in diseases without highly recurrent SNVs. The fact that these two approaches do not rely upon first sequencing a patient’s tumour has advantages for evaluation of ctDNA in large retrospective cohorts, and in multicentre studies where tumour biopsy tissue might not be readily available. Further, such approaches may be generalisable to other tumours that are translocation-driven or characterised by copy number variations.

For patients with these diseases, risk stratification has historically depended on the presence of radiologically detected metastatic disease, and in some instances, primary disease site. Currently, there is no validated tool available at initial diagnosis to identify patients with localised disease at high risk of relapse. While the detection of specific highly recurrent SNVs using ctDNA in pre-treated, colorectal carcinomas has been associated with prognosis,^35^ prior attempts to utilise pre-treatment circulating tumour markers of poor prognosis in Ewing sarcoma have failed to show a consistent association with outcome.^8,9^ The most successful prior attempts to identify circulating prognostic biomarkers in osteosarcoma have utilised microRNA.^14,15^ We demonstrate the potential for ctDNA burden at initial diagnosis to be utilised as a prognostic biomarker of inferior outcomes in these two diseases. Given that in other diseases ctDNA has been associated with stage or disease burden,^36,37^ it is possible that ctDNA levels may be associated with disease burden or occult metastatic disease in the context of these sarcomas. If validated, these assays may improve risk stratification through identification of patients with localised disease at high risk of relapse.

All samples analysed in our study were collected as part of COG biology studies and banked. The samples were collected on these studies without a specific plan for future ctDNA evaluation. Therefore, the collection and handling strategies used in these studies were not ideal for maintaining the integrity of ctDNA samples. That ctDNA was detectable in nearly half of all samples speaks to the robust nature of these assays. Although it will be important to validate these findings in a prospective study, the use of previously banked samples provided the only opportunity to perform a timely evaluation of the prognostic value of ctDNA in these two rare diseases. Furthermore, this study now justified the development of a recently opened prospective study which will optimise sample collection and allow for prospective validation of our findings. Similarly, tumour size, a key clinical feature needed to assess tumour burden, was available only for a subset of patients and was assessed in a non-uniform fashion, potentially limiting the possibility for detecting a strong association with ctDNA positivity. Another limitation of our ctDNA assays is that they were not optimised to identify point mutations in samples with low cancer genome fraction, a problem which may have been compounded by sample quality in the context of this retrospective study. Yet, we could detect mutations in *TP53* and *STAG2* in a limited set of ctDNA Ewing sarcoma samples. We caution that these mutations, particularly mutations in *TP53*, may in fact be heterozygous germline mutations. Such analysis would be more robust with available germline sequencing and serial ctDNA samples, which were not available for these two cohorts of patients.

In summary, the use of two NGS ctDNA assays provides a robust means of detecting ctDNA in the absence of tumour biopsy tissue for two rare and genomically diverse malignancies. Qualitative and quantitative detection of ctDNA in these diseases provides prognostic information that may ultimately be used to improve risk stratification approaches. In order to move this finding into the clinic, we are planning a larger prospective validation study that will also assess the clinical utility of serial ctDNA samples during treatment. We will investigate whether such data could provide an early indication of chemoresponsiveness and serve as a minimal residual disease marker. Finally, with refinement of our assays and improved sample collection, we will further explore the capacity of these two technologies to uncover important tumour characteristics in the peripheral blood, which may provide key information at diagnosis, and inform our understanding of the clonal evolution of these sarcomas during treatment.

## Electronic supplementary material

Supplemental Methods

Supplemental Figure Legends

Supplemental Figure 1

Supplemental Figure 2

Supplemental Figure 3

Supplemental Figure 4

Supplemental Table 1

Supplemental Table 2

## Availability of data and materials

All data are stored locally at the Dana-Farber Cancer Institute and can be made available upon request.
