# Correction: Detection of circulating tumour DNA is associated with inferior outcomes in Ewing sarcoma and osteosarcoma: a report from the Children’s Oncology Group

**DOI:** 10.1038/s41416-019-0424-7

**Published:** 2019-03-18

**Authors:** David S. Shulman, Kelly Klega, Alma Imamovic-Tuco, Andrea Clapp, Anwesha Nag, Aaron R. Thorner, Eliezer Van Allen, Gavin Ha, Stephen L. Lessnick, Richard Gorlick, Katherine A. Janeway, Patrick J. Leavey, Leo Mascarenhas, Wendy B. London, Kieuhoa T. Vo, Kimberly Stegmaier, David Hall, Mark D. Krailo, Donald A. Barkauskas, Steven G. DuBois, Brian D. Crompton

**Affiliations:** 1 Dana-Farber/Boston Children’s Cancer and Blood Disorders Center, Harvard Medical School, Boston, MA USA; 2 Center for Cancer Genome Discovery, Dana-Farber Cancer Institute, Boston, MA USA; 3 Department of Medical Oncology, Dana-Farber Cancer Institute, Boston, MA USA; 4 Broad Institute, Cambridge, MA USA; 5 Center for Childhood Cancer and Blood Diseases at Nationwide Children’s Hospital Research Institute and the Division of Pediatric Heme/Onc/BMT at The Ohio State University, Columbus, OH USA; 6 Department of Pediatrics, MD Anderson Cancer Center, Houston, TX USA; 7 Department of Pediatrics, University of Texas Southwestern Medical Center at Dallas, Dallas, TX USA; 8 Division of Hematology, Oncology, and Blood and Marrow Transplantation, Children’s Hospital Los Angeles, Keck School of Medicine, University of Southern California, Los Angeles, CA USA; 9 Department of Pediatrics, UCSF Benioff Children’s Hospital, University of California, San Francisco School of Medicine, San Francisco, CA USA; 10 Children’s Oncology Group, Monrovia, CA USA; 11 Department of Preventative Medicine, Keck School of Medicine, University of Southern California, Los Angeles, CA USA; 12 Present Address: Department of Pediatric Oncology, 450 Brookline Avenue, Boston, MA USA

**Keywords:** Tumour biomarkers, Sarcoma, Bone cancer

**Correction to:** *British Journal of Cancer* (2018) **119**, 615-621; 10.1038/s41416-018-0212-9; www.bjcancer.com; published online 21 August 2018

The authors have noticed that the final paragraph of the Results section contains errors in the number of patients involved. The correct number of patients is included in the text below. These errors do not affect the Figure referenced.

In osteosarcoma, we focused on 8q gain as a specific biological feature of interest. Among the 41 patients with detectable ctDNA in the osteosarcoma cohort, 8q gain was detected in 73.2% (30/41). The 3-year EFS for patients with 8q gain (*n* = 30) in ctDNA was 60.0% (95% CI 40.5–75.0) compared to 80.8 (95% CI 42.4–94.9) in patients without 8q gain (*n* = 11) in ctDNA (*p* = 0.18; Fig. 3).

