# Supplementary material for: Detection of circulating tumour DNA is associated with inferior outcomes in Ewing sarcoma and osteosarcoma: a report from the Children’s Oncology Group
Source: Br J Cancer. 2018 Aug 21;119(5):615–21. doi: 10.1038/s41416-018-0212-9 (PMC6162271; doi:10.1038/s41416-018-0212-9)
Supplement: Supplementary file 1 — Supplemental Methods [file 41416_2018_212_MOESM1_ESM.docx]

**Supplemental Methods**

DNA was extracted from four slide-mounted FFPE tumor slices using QIAamp DNA FFPE Tissue KIT (Qiagen) and eluted in 100 µL of Buffer ATE (Qiagen). Primers were designed using Primer3Plus (<http://www.bioinformatics.nl/cgi-bin/primer3plus/primer3plus.cgi>) based on the fusion contigs identified by BreaKmer and targeted the identified gene-gene breakpoint and were manufactured by IDT.

*EWS-CSDM2 fusion contig* AAGTTGATTTTTAACTTCCATATTAGCAAATACTCTTCACTACTGAAAGACAGTACTATTTTGCATATTCTGCATAACTTTAAGATTGTACAATTATCTGGAAAGGAGCAGCAACCTTGAAACCTGGGGAGCCAGTTCATTATTCTGATGATTG

EWS-CSMD2 F ACTCTTCACTACTGAAAGACAG

EWS-CSMD2 R AGGTTTCAAGGTTGCTGCTC

RPP30 F AAGAAAGCCAAGTGTGAGGG

RPP30 R AAGAAGGGAGTGCTGACAGA

Primers were validated against synthetic DNA created for the fusion contig (gBlocks, IDT). EW8 cell line DNA was used as a negative control for the fusion specific primers. Primers for the housekeeping gene RPP30 were designed by Bio-Rad. PCR was performed with fusion specific primers, and RPP30 primers with the synthetic DNA control, EW8 cell line, tumor DNA, and a no-template control. Amplification was performed in 200 µL tubes using 22.5 µL of PCR Supermix (Life Technologies), 0.5 µL each of 10 µM forward and reverse primers, and 2 µL template using a Bio-Rad thermal cycler with the following conditions: 5 min at 94°C for initial denaturation, followed by 35 cycles of 30 s at 94°C for denaturation, 30 s at 55°C for annealing, 60 s at 72°C for extension, and 5 min at 72°C for the final extension. PCR products were examined by gel electrophoresis in a 1.25% (w/v) agarose gel in 1 X TAE buffer with 1 X GelRed (Biotium) at 150 V for 50 min and visualized using UV light.
