# Supplementary material for: Detection of circulating tumour DNA is associated with inferior outcomes in Ewing sarcoma and osteosarcoma: a report from the Children’s Oncology Group
Source: Br J Cancer. 2018 Aug 21;119(5):615–21. doi: 10.1038/s41416-018-0212-9 (PMC6162271; doi:10.1038/s41416-018-0212-9)
Supplement: Supplementary file 2 — Supplemental Figure Legends [file 41416_2018_212_MOESM2_ESM.docx]

Supplemental Figure Legends

Supplemental Figure 1 – Correlation between percent ctDNA and cell-free DNA (ng/mL) for all patients with Ewing sarcoma (n=94) with Spearman’s correlation coefficient.

Supplemental Figure 2 - Agarose gel electrophoresis showing amplified products of polymerase chain reaction performed with EWSR1/CSMD2 novel fusion specific primers and RPP30 housekeeping gene control primers tested against a synthetic double-stranded positive control (Synthetic Ctr) created to match the identified novel fusion contig, EW8 cell line DNA, matched tumor DNA from patient with novel EWSR1/CSMD2 detected in cell-free DNA (Tumor DNA), and a no template control. EWSR1/CSMD2 novel fusion specific primers amplified DNA only in the synthetic positive control and in the patient’s tumor.

Supplemental Figure 3 - Correlation between percent ctDNA and cell-free DNA (ng/mL) for all patients with osteosarcoma (n=72) with Spearman’s correlation coefficient.

Supplemental Figure 4A - EFS by ctDNA detection in cohort of patients with metastatic Ewing sarcoma at diagnosis.

Supplemental Figure 4B - Overall survival by ctDNA detection in cohort of patients with metastatic Ewing sarcoma at diagnosis.
