# Supplementary figures and images for: Detection of circulating tumour DNA is associated with inferior outcomes in Ewing sarcoma and osteosarcoma: a report from the Children’s Oncology Group
Source: Br J Cancer. 2018 Aug 21;119(5):615–21. doi: 10.1038/s41416-018-0212-9 (PMC6162271; doi:10.1038/s41416-018-0212-9)

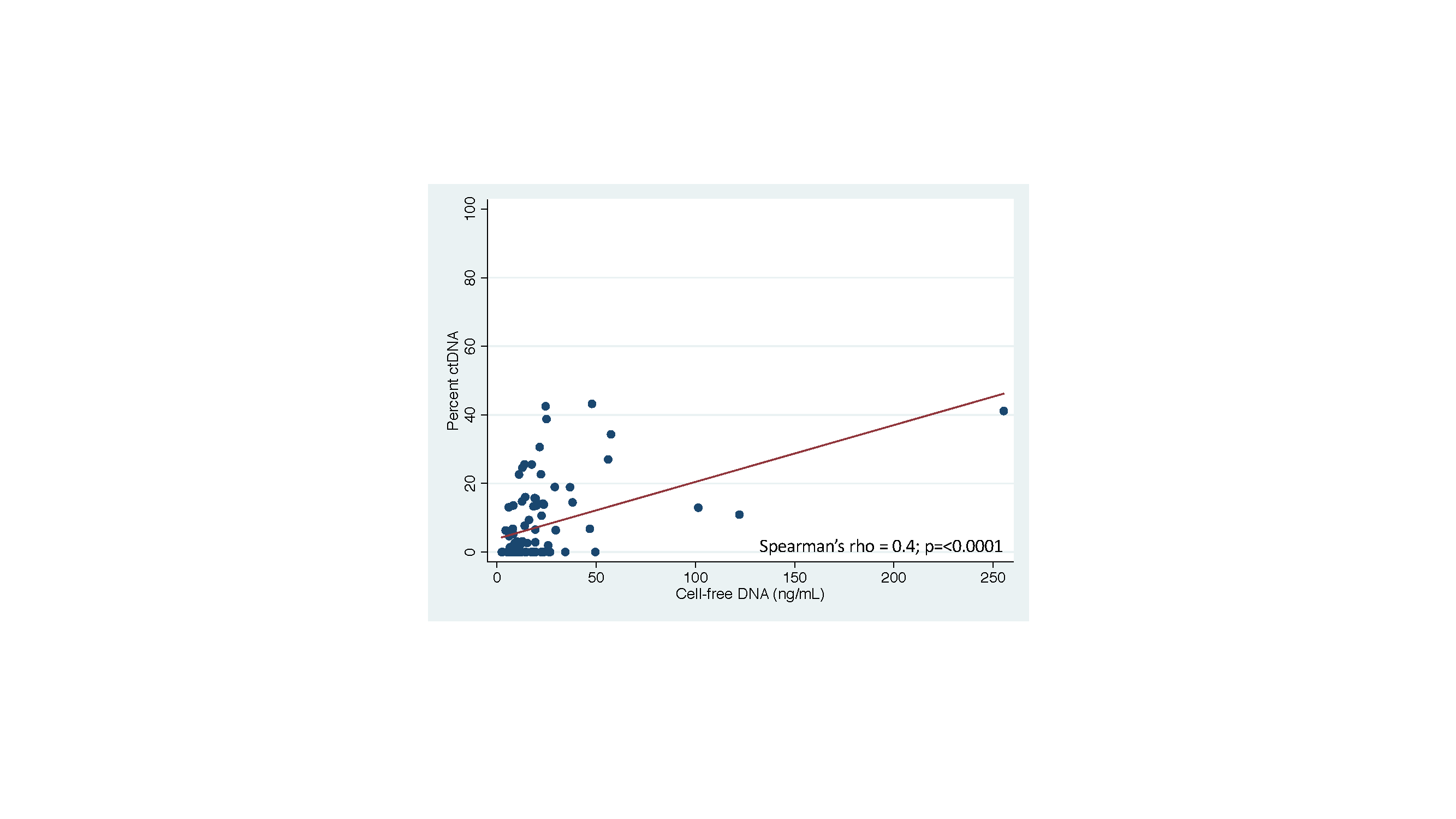

Supplement: Supplementary file 3 — Supplemental Figure 1 [file 41416_2018_212_MOESM3_ESM.tif]

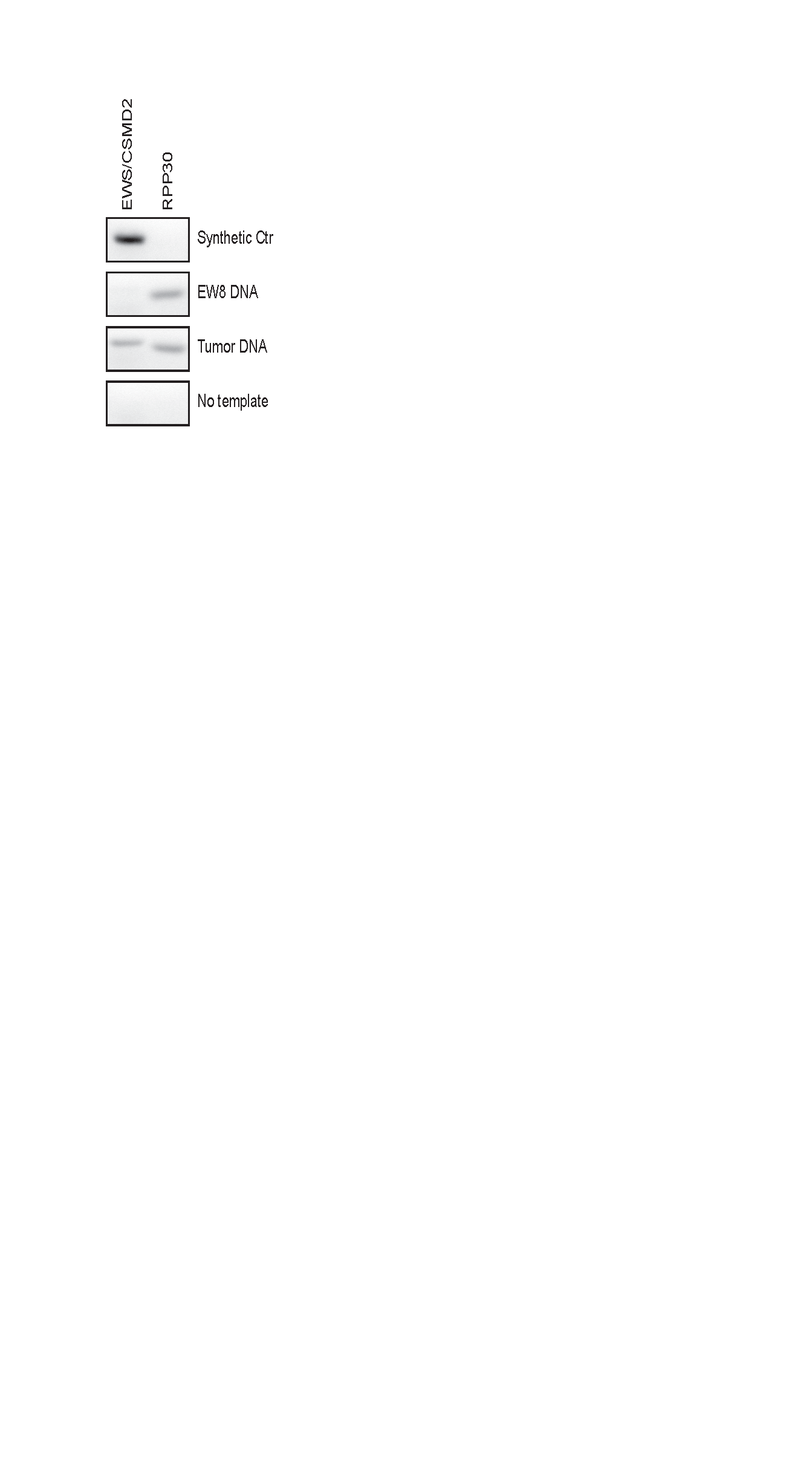

Supplement: Supplementary file 4 — Supplemental Figure 2 [file 41416_2018_212_MOESM4_ESM.tif]

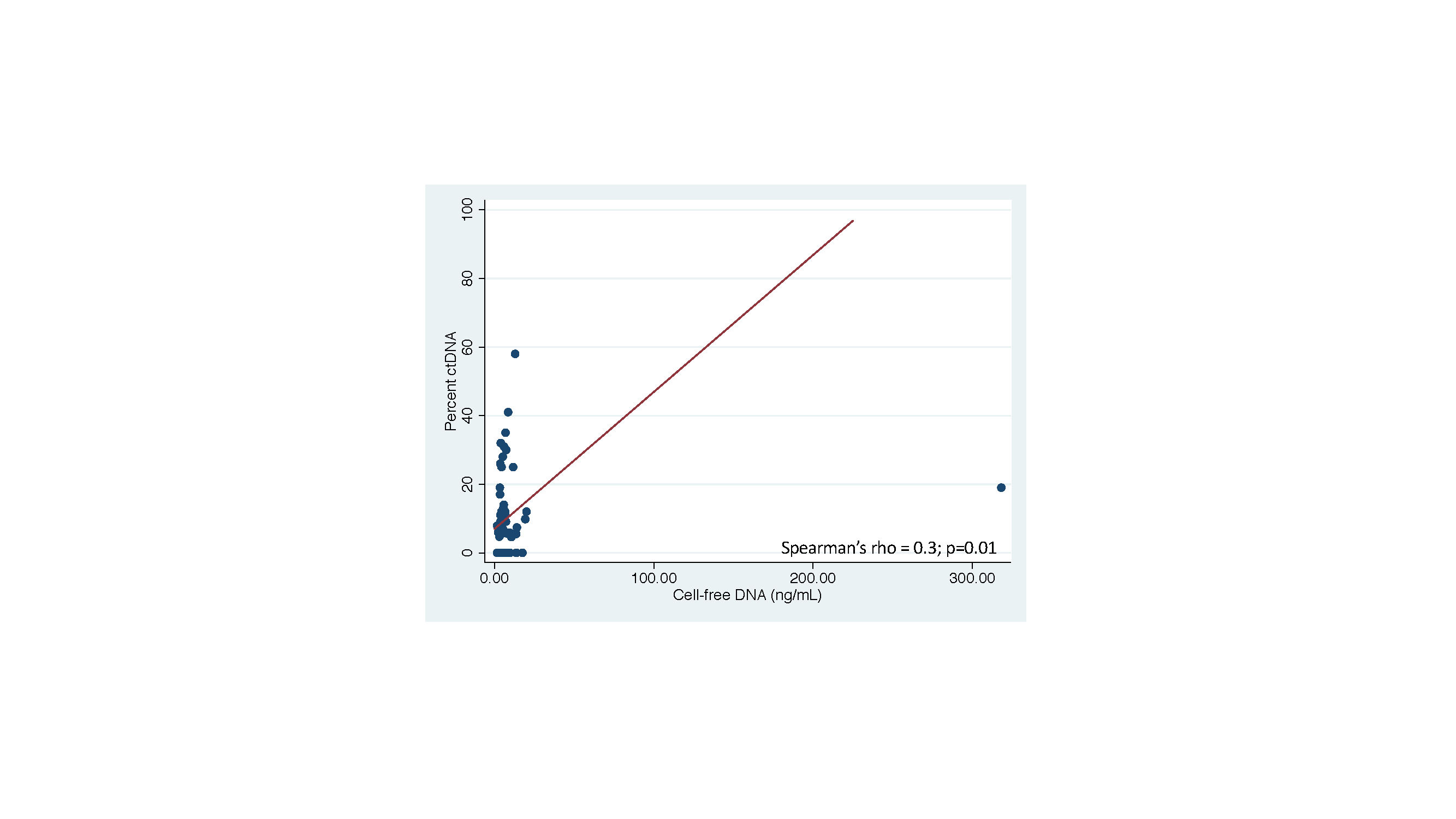

Supplement: Supplementary file 5 — Supplemental Figure 3 [file 41416_2018_212_MOESM5_ESM.tif]

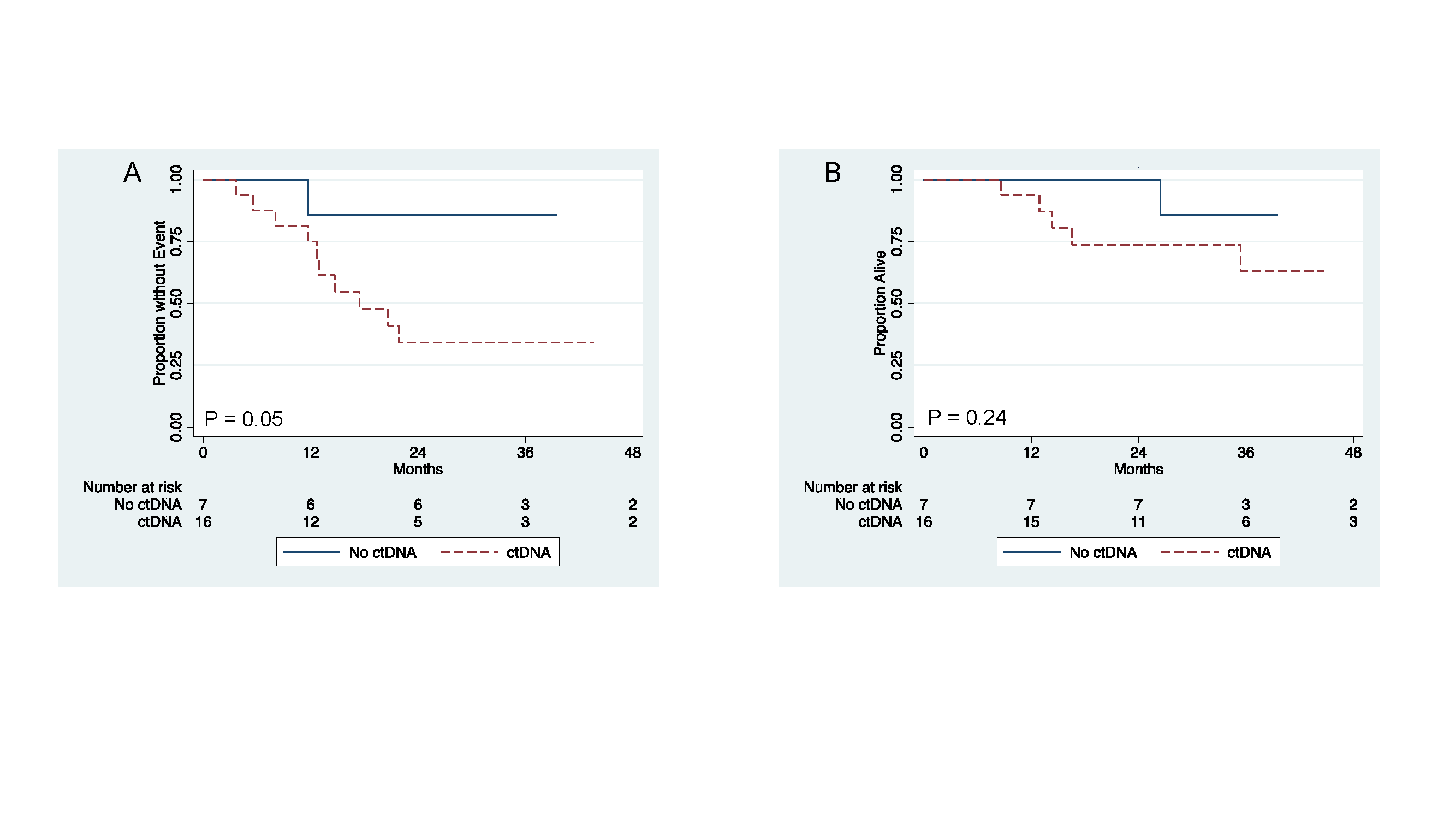

Supplement: Supplementary file 6 — Supplemental Figure 4 [file 41416_2018_212_MOESM6_ESM.tif]
