# Supplementary material for: Detection of circulating tumour DNA is associated with inferior outcomes in Ewing sarcoma and osteosarcoma: a report from the Children’s Oncology Group
Source: Br J Cancer. 2018 Aug 21;119(5):615–21. doi: 10.1038/s41416-018-0212-9 (PMC6162271; doi:10.1038/s41416-018-0212-9)
Supplement: Supplementary file 7 — Supplemental Table 1 [file 41416_2018_212_MOESM7_ESM.docx]

| **Supplemental Table 1.** Association between total cell-free DNA concentration (ng/mL) and clinical features and outcomes in patients with Ewing sarcoma. | | |
| --- | --- | --- |
|  | **Cell-free DNA median (range)** | **p-value** |
| **Ewing sarcoma (n=94)** | | |
| **Entire cohort** | 14.2 ng/mL (2.4 – 255.3) |  |
|  |  |  |
| **Initial Diagnosis (n=77)** | 14.0 ng/mL (2.4 – 101.4) | 0.6 |
| **Relapse (N=17)** | 17.2 ng/mL (5.1 – 255.3) |  |
|  |  |  |
| **Initial Diagnosis (n=77)** |  |  |
| **Age <18 (n = 66)** | 14.1 ng/mL (2.4 – 101.4) | 0.6 |
| **Age > 18 (n = 11)** | 12.8 ng/mL (2.8 – 38.1) |  |
|  |  |  |
| **Male (n = 42)** | 14.3 ng/mL (2.5 – 101.4) | 0.5 |
| **Female (n = 35)** | 12.6 ng/mL (2.4 – 46.8) |  |
|  |  |  |
| **Metastatic (n = 26)** | 15.1 ng/mL (6.6 – 57.5) | 0.5 |
| **Non-metastatic (n = 50)** | 13.7 ng/mL (2.4 – 101.4) |  |
|  |  |  |
| **Pelvic Primary (n = 18)** | 21.2 ng/mL (8.0 – 57.5) | 0.008 |
| **Non-pelvic primary (n = 54)** | 12.5 ng/mL (2.4 – 101.4) |  |
|  |  |  |
| **Tumor size < 8 cm (n = 15)** | 14.2 ng/mL (5.8 – 49.6) | 0.8 |
| **Tumor size ≥ 8 cm (n = 6)** | 12.1 ng/mL (9.0 – 25.0) |  |
|  |  |  |
| **Event-free survival hazard ratio (95% CI) among patients with newly diagnosed localized disease using cell-free DNA concentration as sole covariate** | 1.02 (1.0 - 1.04) | 0.1 |
| **Overall survival hazard ratio (95% CI) among patients with newly diagnosed localized disease using cell-free DNA concentration as sole covariate** | 1.04 (1.01 - 1.08) | 0.01 |
