# Supplementary material for: Detection of circulating tumour DNA is associated with inferior outcomes in Ewing sarcoma and osteosarcoma: a report from the Children’s Oncology Group
Source: Br J Cancer. 2018 Aug 21;119(5):615–21. doi: 10.1038/s41416-018-0212-9 (PMC6162271; doi:10.1038/s41416-018-0212-9)
Supplement: Supplementary file 8 — Supplemental Table 2 [file 41416_2018_212_MOESM8_ESM.docx]

| **Supplemental Table 2.** Association between total cell-free DNA concentration (ng/mL) and clinical features and outcomes in patients with osteosarcoma. | | |
| --- | --- | --- |
|  | **Cell-free DNA median (range)** | **p-value** |
| **Osteosarcoma (n=72)** | | |
| **Mean cell-free DNA concentration (range)** | 4.5 ng/mL (1.7 - 318.2) |  |
|  |  |  |
| **Age < 14 (n = 40)** | 4.7 ng/mL (1.7 – 318.2) | 0.6 |
| **Age > 14 (n = 32)** | 4.5 ng/mL (1.7 – 20.1) |  |
|  |  |  |
| **Male (n = 46)** | 4.4 ng/mL (1.7 - 318.2) | 0.7 |
| **Female (n = 26)** | 4.8 ng/mL (1.7 – 13.0) |  |
|  |  |  |
| **Femur primary (n = 31)** | 5.8 ng/mL (2.0 - 20.1) | 0.06 |
| **Other primary (n = 41)** | 4.0 ng/mL (1.7 – 318.2) |  |
|  |  |  |
| **Event-free survival hazard ratio using cell-free DNA concentration as sole covariate** | 1.01 (1.00 - 1.01) | 0.08 |
| **Overall survival hazard ratio using cell-free DNA concentration as sole covariate** | 0.99 (0.96 - 1.03) | 0.7 |
